# Antiplatelet Therapy for Elderly Patients with Acute Coronary Syndrome Undergoing Percutaneous Coronary Intervention

**DOI:** 10.3390/jcm13144229

**Published:** 2024-07-19

**Authors:** Vincenzo Fioretti, Luca Sperandeo, Donato Gerardi, Aldo Di Fazio, Eugenio Stabile

**Affiliations:** 1 Division of Cardiology, Cardiovascular Department, Azienda Ospedaliera Regionale “San Carlo”, 85100 Potenza, Italy; 2 Department of Advanced Biomedical Sciences, Federico II University of Naples, 80131 Naples, Italy; 3 Regional Complex Intercompany Institute of Legal Medicine, Azienda Ospedaliera Regionale ”San Carlo”, 85100 Potenza, Italy; aldo.difazio@ospedalesancarlo.it

**Keywords:** elderly, antiplatelets, acute coronary syndrome, percutaneous coronary intervention

## Abstract

The elderly represent an increasing proportion of patients presenting with acute coronary syndrome (ACS). Various data have shown that the benefits of percutaneous coronary revascularization are maintained in elderly patients presenting with ACS. Conversely, the management of antiplatelet therapy remains challenging and controversial, because older patients are usually at a high risk of both ischemia and bleeding. Moreover, the recommended ischemic and bleeding risk scores in patients with ACS were developed from studies with a low representation of older patients. New antiplatelet strategies have been developed, but their evidence in elderly patients is limited because they are usually underrepresented in randomized clinical trials due to their clinical complexity. The aim of this review is to summarize the different factors associated with increased ischemic and/or bleeding risk and the scientific evidence about the different antiplatelet strategies in elderly patients presenting with ACS and undergoing percutaneous coronary revascularization.

## 1. Introduction

In the last decades, the improvement of living standards has led to an increase in average life expectancy and, consequently, the progressive ageing of the population. The percentage of the worldwide population aged 65 and above is expected to rise from 10% in 2022 to 16% in 2050 and to 24% in 2100. In 2022, Italy was the European country with the largest share of the elderly population, with 23.8% of the total population aged 65 years and older. Globally, the number of people aged 80 years or over is rising even faster than the number aged 65 or above. By 2050, the world will have an estimated 459 million people aged 80 or more, almost three times the number in 2021 [1]. In the last years, the increased life expectancy has led some geriatric societies to propose raising the threshold that defines a person as elderly from 65 to 75 years old [2]. The incidence of cardiovascular diseases increases with age, from about 40% in adults aged 40–59 years to 75% in those aged 60–79 years and 86% in those older than 80 years [3]. The elderly population constitutes an increasingly larger proportion of the patients admitted for acute coronary syndrome (ACS); in fact, patients aged 75 years or older constitute about one-third of the hospitalizations of patients admitted with ACS [4]. Older age has been found to be a predictor of the lower use of cardiac catheterization [5], despite the fact that early myocardial revascularization has proven to be superior to a conservative strategy also used in this category of patients [6,7]. Similarly, antiplatelet therapy in elderly patients with ACS remains a debated topic due to the difficulty involved in balancing the risk of ischemic and bleeding events [8].

## 2. Thrombotic and Bleeding Risk in Elderly Patients

Antiplatelet therapy is the mainstay of the medical treatment in patients with ACS. The goal of antiplatelet therapy is to reduce the risk of the recurrence of ischemic events after coronary revascularization; however, it increases the risk of bleeding events [9]. A large metanalysis including patients with coronary artery disease (CAD) showed that major and late bleedings are associated with a similar increase in mortality to that of myocardial infarction, whereas early bleeding might have a stronger association with mortality [10]. Therefore, it is important to evaluate the thrombotic and bleeding risk for every patient in order to choose the optimal antiplatelet strategy, in terms of composition and duration [11].

**Figure 1:**
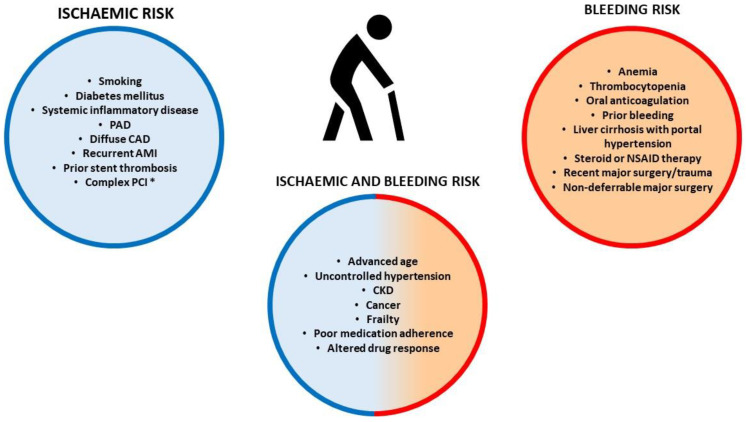
Factors associated with increased ischemic and/or bleeding risk. AMI: acute myocardial infarction; NSAID: non-steroidal anti-inflammatory drugs; * Complex PCI: 3 vessels treated, ≥3 stents implanted, total stent length > 60 mm, left main, bifurcation stenting with ≥2 stents implanted, chronic total occlusion, stenting of last patent vessel [22].

Ageing is a non-modifiable risk factor for both ischemic and bleeding events [12]. Endothelial dysfunction, blood stasis, and increased platelet reactivity may contribute to the enhanced thrombotic risk in older patients [13]. The common comorbidities of older patients, like diabetes, smoking, and concomitant peripheral artery disease (PAD), contribute to the increased ischemic risk (Figure 1). Moreover, elderly patients often present with more complex CAD, multivessel disease, challenging anatomy, and a higher burden of coronary calcium deposits [14]. 

In contrast, age-related collagen and amyloid deposits in the arterial wall weaken the vessel, predisposing to bleeding [15]. However, the Academic Research Consortium for High Bleeding Risk (ARC-HBR) indicated age ≥ 75 years as a minor criterion to identify high bleeding risk (HBR) patients [16]. In fact, bleeding risk increases with age with some confounding resulting from comorbidities, which tend to cumulate in elderly patients. Therefore, it must be acknowledged that chronological age does not always reflect biological age [17]. The common comorbidities of older patients, like anemia, thrombocytopenia, history of bleeding, and long-term use of oral non-steroidal anti-inflammatory drugs or steroids, contribute to increased bleeding risk. 

However, some comorbidities, like uncontrolled hypertension, chronic kidney disease (CKD), cancer, and prior ischemic stroke, can further increase both bleeding and thrombotic risk [8]. Atrial fibrillation is another common comorbidity in older patients, and its incidence rises with increasing age, as does the associated risk of embolic stroke. Oral anticoagulation (OAC) reduces the risk of embolic stroke, although it increases the risk of bleeding [18]. Moreover, changes in organ function, poor medication adherence, and polypharmacy-related drug interactions can influence pharmacokinetic and pharmacodynamic responses to antithrombotic drugs [19]. Also, frailty, a clinical condition characterized by an increased vulnerability to adverse outcomes when exposed to stressors, can influence both the ischemic and the bleeding risk in elderly patients [20,21]. Therefore, physicians should assess the thrombotic and the bleeding risk of each patient by considering clinical, anatomical, procedural, and laboratory data. 

To standardize the assessment of the ischemic risk, the European Society of Cardiology (ESC) guidelines recommend evaluating clinical factors and technical aspects of the percutaneous coronary intervention (PCI) [22]. In the same way, to standardize the assessment of the bleeding risk, the ESC guidelines recommend using ARC-HBR criteria, a list of clinical and biochemical data that identify HBR as a risk of major bleeding of ≥4% or a risk of intracranial hemorrhage of ≥1% at one year [16]. Alternatively, the PRECISE-DAPT score identifies HBR patients using five variables, including age, prior bleeding, hemoglobin, white blood cells, and creatinine clearance [23]. Age is one of the main predictors of bleeding in the PRECISE-DAPT series, accounting for a significant proportion of the score. In fact, most elderly patients have PRECISE-DAPT values above the recommended cut-off point for bleeding risk [24]. However, recommended bleeding risk scores in patients with ACS were developed from a series with a low representation of older patients. In a retrospective analysis of a single-center registry, including only elderly patients, the PRECISE-DAPT score and the ARC-HBR criteria showed insufficient predictive value for predicting bleedings, while a truly simplified clinical evaluation, consisting of only three binary clinical variables (hemoglobin < 11 g/100 mL, previous bleeding, and anticipated use of anticoagulants), provided equal discriminatory potential, but also demonstrated superior predictive value, as determined by Cox regression models [25].

The DAPT score evaluates the combined ischemic and bleeding risk, and it is recommended for use in patients who tolerated 12 months of dual antiplatelet therapy (DAPT) to select those eligible for treatment prolongation. Advanced age is the main predictor of bleeding in the score; in fact, patients with 65 to 74 years of age and ≥75 years of age have −1 and −2 points, respectively [26]. In a prospective study, including only older Chinese patients treated with PCI, the DAPT score did not show predictive value for 5-year ischemic events; however, it had a certain predictive value for 5-year BARC (Bleeding Academic Research Consortium), 5-year BARC (Bleeding Academic Research Consortium) ≥ 2 bleedings, especially in patients with higher values of the score [27].

## 3. Antithrombotic Strategies in Elderly Patients Not Requiring Anticoagulation

### 3.1. Standard DAPT: Which P2Y12 Inhibitor to Choose?

As for younger patients, 12 months of DAPT with acetylsalicylic acid (ASA) and a P2Y12 receptor inhibitor is also the recommended antiplatelet strategy in elderly patients after PCI to reduce the risk of stent thrombosis and target lesion failure, unless there is HBR [28]. According to the current guidelines, a potent P2Y12 inhibitor like ticagrelor or prasugrel should be preferred, while clopidogrel is recommended when ticagrelor and prasugrel are not available, cannot be tolerated, or are contraindicated. However, the choice of the P2Y12 receptor inhibitor in elderly patients is debated due to their increased bleeding risk. The effects of different P2Y12 inhibitors in elderly patients have been assessed in subgroup analyses of pivotal trials and dedicated age-specific studies (Table 1). The CURE (Coronary Intervention-Clopidogrel in Unstable Angina to Prevent Recurrent Events) trial evaluated the addition of clopidogrel to ASA, compared to ASA alone, in patients with NSTE-ACS. Almost half the population were aged 65 years or older, but only about 21.2% of the study population were treated with PCI. After 12 months, clopidogrel reduced the incidence of the composite of death from cardiovascular causes, non-fatal myocardial infarction, or stroke, but with an increase in major bleedings compared with a placebo [29]. Data from the SWEDEHEART registry (Swedish Web System for Enhancement and Development of Evidence-Based Care in Heart Disease Evaluated According to Recommended Therapies), including more than 14,000 patients aged 80 years or older with myocardial infarction, showed that clopidogrel was the most prescribed P2Y12 receptor inhibitor (clopidogrel 60.2%, ticagrelor 39.8%) [30]. However, the elderly population present a high rate of impaired response to clopidogrel, with high platelet reactivity compared with younger patients [31]. Conversely, the newer-generation P2Y12 receptor inhibitors, ticagrelor and prasugrel, have a more potent and predictable action with more effective prevention of recurrent ischemic events after ACS. However, such a benefit comes at the price of increased bleeding risk [32]. Among the newest P2Y12 receptor inhibitors, prasugrel at the standard dose of 10 mg daily in the TRITON-TIMI 38 trial (Trial to Assess Improvement in Therapeutic Outcomes by Optimizing Platelet Inhibition with Prasugrel–Thrombolysis in Myocardial Infarction study) did not show a significant clinical benefit compared with clopidogrel, with an increase in major bleedings in ACS patients aged 75 years or older (about 13% of the study population) undergoing PCI [33]. For that reason, prasugrel at the standard dosage is not recommended for elderly patients by the European Medicines Agency. A reduced dose of prasugrel (5 mg daily) showed similar thrombotic and bleeding events compared to clopidogrel in the ELDERLY ACS-2 study, which included only patients aged 75 years or older undergoing PCI for ACS [34]. In a sub-analysis of the ISAR-REACT 5 trial (Intracoronary Stenting and Antithrombotic Regimen: Rapid Early Action for Coronary Treatment), a reduced dose of prasugrel, compared with the standard dose of ticagrelor in elderly or low-weight patients with ACS, was associated with maintained anti-ischemic efficacy while protecting these patients against the excess risk for bleeding [35,36]. Treatment with ticagrelor significantly reduced the rate of ischemic events, but with an increase in the rate of major bleedings not related to coronary artery bypass grafting (more cases of fatal intracranial bleeding and fewer of fatal bleeding of the other types) when compared with clopidogrel in the PLATO trial (the Study of Platelet Inhibition and Patient Outcomes), which included about 15% ACS patients aged 75 years or older, of whom about 45% were undergoing planned PCI [37]. A sub-study of the PLATO trial showed that the significant clinical benefit and overall safety of ticagrelor compared with clopidogrel was independent of age [38]. An analysis of the SWEDEHEART register, which enrolled more than 14,000 patients aged 80 years or older with myocardial infarction, of whom only about 13% were undergoing PCI, showed that the use of ticagrelor was associated with an increased risk of bleeding and death compared with clopidogrel [30]. More recently, the POPular AGE study randomized NSTE-ACS patients aged 70 years or older to clopidogrel or ticagrelor or prasugrel; of these patients, about 47% were undergoing PCI. The results showed a significant reduction in bleeding events in patients treated with clopidogrel without an increase in the combined endpoint of death, myocardial infarction, stroke, and bleeding [39]. In a network metanalysis including 14,485 patients aged >70 years, prasugrel was associated with the highest probability of a reduction in ischemic events and clopidogrel of bleedings [40]. A large meta-analysis that included 29,217 elderly patients presenting with both ACS-STEMI and NSTEMI, confirmed the efficacy and safety of clopidogrel when compared with ticagrelor and prasugrel [41]. Due to this evidence, the latest ESC guidelines recommend that older patients, especially those with HBR, consider clopidogrel as the P2Y12 receptor inhibitor in the DAPT with ASA [22].

### 3.2. Alternative Strategies

The incidence of ischemic events is highest during the first month after PCI and then tends to reduce gradually. In contrast, the risk of bleeding during DAPT, despite being relatively high in first few days after PCI due to the use of an arterial access site, does not diminish over time. Therefore, the net benefit of DAPT might decrease over time [9]. For that reason, alternative strategies to reduce the bleeding risk correlated with DAPT have been tested. 

#### 3.2.1. Shortening DAPT

Older patients and patients with HBR used to receive bare-metal stents (BMS) instead of drug-eluting stents (DES) to shorten the duration of DAPT and to reduce the risk of the bleeding complications associated with prolonged antithrombotic therapy. The newer generations of DES, due to thinner struts, no polymer or a biocompatible polymer, faster re-endothelization, and low rates of thrombosis, allowed the consideration of a progressive shortening of the standard DAPT regimen, especially in HBR patients [42]. The SENIOR trial included only patients aged 75 years or older undergoing PCI; these patients were randomized to the implantation of a BMS or a newer-generation DES, followed by a short BMS-like DAPT regimen (6 months in patients with ACS). The results at one year showed that DES implantation was associated with a significant reduction in the composite endpoint, including all-cause mortality, myocardial infarction, stroke, or ischemia-driven target lesion revascularization, and with comparable low bleeding rates in both groups [43]. Later, different trials evaluated abbreviated DAPT regimens followed by ASA or P2Y12 inhibitor monotherapy in patients treated with PCI using DES [44], but few randomized trials have been conducted in elderly patients (Table 2) [45,46,47,48,49,50,51]. Among these, the MASTER DAPT study enrolled the largest number of elderly patients with HBR (69% aged ≥75 years); these patients were randomized to 1-month DAPT versus standard DAPT (≥6 months for patients without OAC indication), followed by a single antiplatelet agent (either ASA or, in two-thirds of the patients, a P2Y12 inhibitor) after PCI. Clopidogrel was the most frequently used P2Y12 inhibitor in the standard therapy group and was the most frequently used monotherapy in the abbreviated therapy group at the time of randomization and thereafter. The abbreviated DAPT was non-inferior to the standard therapy in preventing net adverse clinical events and major adverse cardiac or cerebral events [49]. The Xience Short DAPT program comprised three prospective single-arm studies, including 3,652 HBR patients treated with a short DAPT course, followed by ASA monotherapy after PCI. One month of DAPT, compared with three months of DAPT, was associated with similar ischemic outcomes and a lower bleeding risk [52]. The results were also confirmed in older patients aged ≥ 75 years, about 61.4% of the study population; of this population, about 34.3% had ACS [53]. A network meta-analysis that included 14 trials evaluated various DAPT durations following PCI in 19,102 older adults aged ≥65 years, of whom about 60% had ACS. No differences in net adverse clinical events and major adverse cardiovascular events were seen for 1, 3, 6, and 12 months of DAPT. However, 1 and 3 months of DAPT were both associated with a lower risk of bleeding compared with 6 months of DAPT; in addition, 3 months of DAPT was associated with a lower risk of bleeding compared with 12 months of DAPT [54].

According to the latest ESC guidelines, an abbreviated DAPT duration of one month may be considered in HBR patients, while a longer DAPT duration of 3–6 months should also be considered in patients who are not HBR or in patients who have a high ischemic risk [22]. According to the consensus statement from an international expert panel on coronary thrombosis, single antiplatelet therapy (SAPT) with a P2Y12 inhibitor should be considered after 1–3 months of DAPT in patients with HBR (clopidogrel is the most studied P2Y12 inhibitor in this setting) or in those without risk factors for bleeding and without high long-term ischemic risk (ticagrelor is the most studied P2Y12 inhibitor in this setting), while SAPT with ASA should be considered after 3–6 months of DAPT, ideally only if the patient is HBR [44]. 

#### 3.2.2. De-escalation Strategy

De-escalation involves downgrading from a potent P2Y12 inhibitor at conventional doses to either clopidogrel or reduced-dose prasugrel in order to reduce the bleeding risk. This strategy could be unguided or guided by platelet reactivity tests [44]. The effects of the de-escalation strategy in elderly patients have been evaluated in subgroup analyses of pivotal trials and dedicated age-specific studies (Table 3) [55,56,57,58,59]. Among the studies evaluating unguided de-escalation, the TALOS-AMI trial (Ticagrelor vs. Clopidogrel in Stabilized Patients With Acute Myocardial Infarction) enrolled Asian patients with ACS undergoing PCI; of these patients, only about 12% were aged ≥75 years. After PCI, each patient received DAPT therapy with ASA plus ticagrelor for one month. After one month without ischemic or bleeding events, the patients were randomized to continue DAPT with ticagrelor or unguided de-escalation (DAPT with clopidogrel). At one year, the unguided de-escalation strategy significantly reduced the risk of net clinical events, mainly by reducing the bleeding events independently of age [55].Among the studies evaluating guided de-escalation, the TROPICAL ACS trial demonstrated that de-escalation guided by a platelet reactivity test was non-inferior to standard treatment with prasugrel at 1 year after PCI in terms of net clinical benefit [56]. However, an age analysis showed a significant net clinical benefit of guided de-escalation in younger patients, while in elderly patients over 70 years of age (about 14% of the study population), the absolute risk of events was higher without significant differences between the guided de-escalation group and the control group [57]. The ANTARCTIC trial is the only study of platelet function testing that exclusively includes elderly patients. The study evaluated a de-escalation strategy guided by platelet reactivity tests in ACS patients aged ≥75 years undergoing PCI. After 2 weeks of DAPT with prasugrel 5 mg, a platelet reactivity test was performed in the experimental group, and based on these results, an evaluation was made regarding whether to increase the dose of prasugrel to 10 mg or switch to clopidogrel. At one year of follow-up, no significant differences were found for either the ischemic or the bleeding events between the two strategies [58]. In the POPULAR GENETIC trial, the de-escalation strategy was guided by genetic testing to evaluate the polymorphisms of the CYP2C19 gene [59]. Clopidogrel, in fact, is a prodrug and its activation occurs in the liver via cytochrome P450 enzymes. In 30% of white patients, an inadequate response to clopidogrel occurs when measured with platelet function tests [60]. This reduction in functionality may be partly explained by genetic variations involving the cytochrome gene [61]; in fact, in patients without these genetic variations, clopidogrel has similar efficacy to ticagrelor and prasugrel [62]. In the POPULAR GENETIC trial, patients with STEMI-ACS undergoing primary PCI (only about 15% were aged ≥75 years) were randomized to a standard treatment with ticagrelor or prasugrel, or to a genotype-guided treatment. After 12 months, the genotype-guided strategy for the selection of oral P2Y12 inhibitor therapy was non-inferior to the standard treatment with ticagrelor or prasugrel for thrombotic events and resulted in a lower incidence of bleeding [59]. A large meta-analysis of 16 studies, which included 47,911 elderly patients aged ≥65 years with ACS undergoing PCI, investigated different DAPT strategies and confirmed the efficacy and safety of the de-escalation strategy, with an improved net clinical benefit compared with DAPT using potent P2Y12 inhibitors [63]. 

According to the current ESC guidelines, the de-escalation strategy may be considered in patients with HBR, but it is not recommended in the first month after the index event [22].

### 3.3. Long-Term Antithrombotic Strategies

After the standard 12 months of DAPT, the current guidelines recommend continuing with ASA in all patients without contraindications for long-term treatment, but different antithrombotic strategies have been assessed (Table 4). An alternative to long-term treatment with ASA is represented by P2Y12 inhibitors [22]. In the HOST-EXAM trial, in which 43% of the included patients were aged ≥65 years, clopidogrel monotherapy compared with ASA monotherapy during the chronic maintenance period after PCI significantly reduced the risk of composite of all-cause death, non-fatal myocardial infarction, stroke, readmission due to ACS, and major bleedings [64]. The age-specific subgroup analysis also confirmed the beneficial effect of clopidogrel over ASA in the elderly group [65]. 

Adding a second antithrombotic agent to ASA for extended long-term secondary prevention should be considered in patients with a high ischemic risk and without HBR [22]. In the DAPT trial, patients undergoing PCI (about 10% of the study population were aged ≥75 years) were randomized after 1 year to maintain, as an adjunct to ASA, a P2Y12 inhibitor (clopidogrel 65%; prasugrel 35%) for 30 months, versus ASA only. Prolonged DAPT significantly reduced the risks of stent thrombosis and major adverse cardiovascular and cerebrovascular events but was associated with an increased risk of bleeding that increased with age. [66]. These findings led to an unfavorable impact of age on the DAPT score, which was developed to identify patients who benefited from extended DAPT [26]. The PEGASUS-TIMI 54 trial (Prevention of Cardiovascular Events in Patients with Prior Heart Attack Using Ticagrelor Compared to Placebo on a Background of Aspirin–Thrombolysis in Myocardial Infarction 54) evaluated prolonged DAPT with ticagrelor in patients with prior myocardial infarction with high risk features, including age ≥65 years. The DAPT with ticagrelor 60 mg b.i.d. significantly reduced the risk of cardiovascular death, myocardial infarction, or stroke and increased the risk of major bleeding. The efficacy regarding the reduction in the ischemic endpoint was also confirmed in patients aged ≥75 years, about 14% of the total population included in the trial; however, the rate of major bleeding events was higher in this group of the population [67]. However, in the PRODIGY trial a shorter duration of clopidogrel-based DAPT (6 months), compared with prolonged therapy (24 months), was associated with a reduction in bleeding risk without a significant difference in the rate of ischemic events in elderly patients undergoing PCI, of whom about 80% presented with ACS [68].

An alternative long-term strategy is represented by the dual antithrombotic therapy (DAT) that includes Rivaroxaban 2.5 mg b.i.d. plus ASA. As for long-term DAPT, the current guidelines suggest DAT for patients with high ischemic risk and without HBR [22]. In the COMPASS trial, among patients with stable atherosclerotic vascular disease, those assigned to DAT therapy had better cardiovascular outcomes and more major bleeding events than those assigned to ASA alone. The efficacy regarding the reduction in the ischemic endpoint was also confirmed in patients aged ≥75 years, about 20% of the total population included in the trial; however, the rate of major bleeding events was higher in this group of the population [69].

**Table 4:** Age-specific data in randomized studies about alternative long-term antithrombotic strategies.

Single Antiplatelet						
Study	ACS Presentation (%)	PCI Rate (%)	Elderly Patients (%)	Groups	Follow-Up (m)	Main Results
HOST EXAM [64,65]	STEMI (17.1%), NSTE-ACS (54.9%)	100%	≥65 y (43.4%)	Clopidogrel vs. ASA	24	Clopidogrel compared to ASA monotherapy significantly reduced the risk of ischemic events and major bleedings. No significant influence by age.
Long DAPT						
DAPT [66]	STEMI (10.5%), NSTE-ACS (32.2%)	100%	≥75 y (10.4%)	ASA + P2Y12i (clopidogrel 65%; prasugrel 35%) vs. ASA only	30	DAPT, compared with aspirin therapy alone, significantly reduced the risks of ischemic events, but with an increased risk of bleeding. The efficacy benefit of prolonged DAPT was attenuated by age and bleeding rates increased with age.
PEGASUS-TIMI 54 [67]	STEMI (53.5%), NSTE-ACS (40.5%)	83%	≥75 y (14.6%)	Ticagrelor 90 mg + ASA vs. ticagrelor 60 mg + ASA vs. ASA + placebo	36	Ticagrelor significantly reduced the risk of ischemic events and increased the risk of major bleeding. The efficacy was also confirmed in elderly patients but with an increased risk of bleeding.
DAT						
COMPASS [69]	History of myocardial infarction (62.2%)	53.9%	≥75 y (20.9%)	Rivaroxaban 2.5 mg + ASA vs. ASA + placebo	36	Rivaroxaban plus ASA reduced the rate of ischemic events with increased risk of bleeding compared with ASA alone.. Despite no significant interaction with age, among elderly patients the magnitude of benefit with DAT was reduced, and the relative increase in major bleeding was higher.

m = months, y = years.

## 4. Antithrombotic Strategies in Elderly Patients Requiring Anticoagulation

AF is one of the most frequently encountered indicators of anticoagulation in elderly patients. AF is the most common arrhythmia, and its incidence rises with advancing age [18]. AF is a comorbidity encountered in approximately 10% of STEMI patients and 13% of NSTE-ACS patients aged ≥75 years [70]; moreover, a new onset of AF is one of the most frequent complications of ACS [71]. Antithrombotic therapy in patients with AF requiring OAC and presenting with ACS is a clinical conundrum due to the increased bleeding risk, especially in elderly patients [72]. After PCI, a period of triple antithrombotic therapy (TAT), including DAPT plus OAC, followed by DAT including SAPT plus OAC, should be prescribed. Clopidogrel is the P2Y12 inhibitor of choice during TAT therapy. The use of ticagrelor or prasugrel as part of TAT is not recommended due to the excessive bleeding risk [22]. According to the individual ischemic and bleeding risks, TAT should be prescribed from one week to one month, and then the subsequent DAT should be continued up to the sixth or twelfth month. At the end of treatment with DAT, it is recommended to interrupt the antiplatelet therapy and to continue only with OAC [22]. In a large metanalysis, which included patients with AF undergoing PCI or with ACS, DAT, particularly when based on NOAC and a P2Y12 inhibitor, was associated with a reduction in bleeding when compared with TAT. This benefit was, however, counterbalanced by a higher risk of cardiac events, mainly stent-related, but not cerebrovascular events [73]. However, in the four pivotal trials comparing DAT and TAT, which were included in the metanalysis, older patients were underrepresented; they represented about one-third in PIONEER AF-PCI [74] and in RE-DUAL PCI [75] and were not reported in AUGUSTUS [76] and ENTRUST AF-PCI [77]. The more recent MASTER-DAPT trial included 4,579 patients with HBR, of whom about 36% had an indication for OAC therapy. The patients with an OAC indication (median age 73 years, 42% with ACS), after 1 month of TAT, were randomized to an abbreviated regimen (interruption of DAPT and SAPT for 5 months) or a standard regimen (DAPT for ≥3 months, followed by SAPT until the 12th month). The rate of net adverse clinical outcomes and major adverse cardiac events did not differ with the abbreviated regimen in patients with HBR with or without OAC and resulted in lower bleeding rates in patients without OAC indication [49].

## 5. Intravenous Antiplatelet Therapies in Elderly Patients

### 5.1. Cangrelor

Cangrelor is the only intravenous P2Y12 receptor inhibitor available for patients with CAD undergoing PCI who had not received an oral P2Y12 inhibitor before the procedure. The latest ACS guidelines also reevaluated the role of pretreatment with oral P2Y12 inhibitor in patients with STEMI [22]. The rationale of pretreatment is to reduce the rate of ischemic events while waiting for invasive treatment, to prevent early-stent thrombosis and to reduce glycoprotein (Gp) IIb/IIIa bail-out use [78]. However, this strategy may unnecessarily expose the patient to an increased bleeding risk if CAD is ultimately not confirmed or if an urgent surgical procedure (e.g., coronary aortic bypass graft or repair of aortic dissection) is needed. Unlike other P2Y12 inhibitors, cangrelor is an intravenous drug that allows rapid, potent, and rapidly reversible inhibition of platelet aggregation and therefore may be considered in P2Y12 receptor inhibitor-naïve patients [79]. Few studies have evaluated cangrelor in elderly patients [28]. In the ARCANGELO study (itAlian pRospective Study on CANGrELOr), only 68 patients aged ≥75 years, about 21% of study population, were treated with cangrelor [80]. A sub-analysis of the CHAMPIONS Phoenix trial evaluated the outcomes of cangrelor use in patients aged ≥75 years (about 18% of the study population). Cangrelor, when compared with clopidogrel, provides similar efficacy in patients ≥ 75 years old to that in those <75 years old but does not increase the risk of major bleedings [81]. Conversely, in a sub-analysis of the ICARUS registry, in which about 32% of enrolled patients were aged ≥75 years, the use of cangrelor was associated with higher rates of net adverse clinical events at 48 h in elderly patients. Moreover, in multivariable analysis, advanced age was an independent predictor of net adverse clinical events [82]. According to the current ACS guidelines, cangrelor may be considered in P2Y12 receptor inhibitor-naïve patients undergoing PCI to reduce the risk of periprocedural myocardial infarction, repeat coronary revascularization, and stent thrombosis. The pharmacokinetics of this drug make it particularly useful in patients for whom it may not be feasible to give oral drugs in the setting of emergent PCI (e.g., patients with cardiogenic shock and/or patients on mechanical ventilation) [22].

### 5.2. Glycoprotein IIb/IIIa Receptor Inhibitors

Gp IIb/IIIa inhibitors prevent platelet aggregation by blocking glycoprotein IIb/IIIa receptors on their platelet’s plasma membrane and inhibiting fibrinogen binding [83]. The available GP IIb/IIIa inhibitors include abciximab, tirofiban, and eptifibatide. The data on its efficacy and safety in older patients are contrasting [28]. The treatment with abciximab also led to an absolute reduction in death, non-fatal myocardial infarction, and repeat revascularization in elderly patients undergoing PCI [84,85]. Conversely, a large observational registry that included more than 800 elderly patients, found no benefit of abciximab treatment in high-risk ACS [86]. However, as a result of perceived bleeding risks, older patients receive GP IIb/IIIa inhibitors less often than younger subjects. In fact, their use was associated with an increased risk of and need for transfusion in octogenarian patients treated with PCI [87]. Conversely, other studies did not show significant bleeding in elderly patients treated with Gp IIb/IIIa [84,85]. According to the current guidelines, GP IIb/IIIa should be considered if there is evidence of no-reflow or a thrombotic complication during PCI [22].

## 6. Medico-Legal Implications

Most of the recommendations about antiplatelet therapy in elderly patients are obtained by the main randomized studies, in which older patients are underrepresented. Due to lack of scientific evidence about the management of antithrombotic therapy in elderly patients with ACS undergoing PCI, few specific recommendations are present in the current ACS guidelines. From a medico-legal perspective, it is pertinent to note that Italian law n. 24/2017, commonly called the Gelli–Bianco law, stipulates in Article 5 the obligation for healthcare professionals to adhere to the guidelines outlined by the Italian National Institute of Health. In the absence of these guidelines, adherence to good clinical and health practices is mandatory. Article 6 of the same law also provides immunity from punishment for personal injury and medical malpractice if the event resulted from incompetence and the prescribed guidelines or good clinical practices were duly followed, provided that these directives are suitable for the specific circumstances at hand [88]. It is essential to recognize that guidelines serve as a benchmark for evaluating the conduct of healthcare professionals and do not function as binding regulations. In the case that the recommendations prove inadequate for addressing the specific requirements of a given case, healthcare professionals are expected to exercise discretion and to deviate from them. This is particularly relevant in the context of elderly patients, who commonly present with complex medical conditions and are subjected to polypharmacy. In such instances, the management of geriatric patients may not align with the guidelines designed for single pathologies. Furthermore, it is important to acknowledge that the studies underpinning these recommendations often involve “ideal” patients and may not encompass the nuances associated with fragile patients. Consequently, in situations of this nature, the autonomy of healthcare professionals is paramount, as they are tasked with evaluating, based on scientific evidence, the appropriateness of the diagnostic and therapeutic decisions recommended by the guidelines.

## 7. Discussion

Ageing and associated comorbidities influence both the ischemic and the bleeding risk. As a consequence, elderly patients are often underrepresented or excluded in randomized trials [89,90]. The guidelines support the use of risk scores to guide the choice of the composition and duration of antithrombotic therapy [22], although these tools were developed from studies with a low representation of older patients. Moreover, recommended risk scores do not take the frequent conditions of the elderly into consideration, like frailty, changes in organ function, poor medication adherence, and polypharmacy-related drug interactions, that could influence both the ischemic and the bleeding risk. Due to the complexity of geriatric patients, the assessment of the ischemic and bleeding risks should not be limited to the in-hospital stay, but it should require frequent reassessments during the follow-up period. 

**Figure 2:**
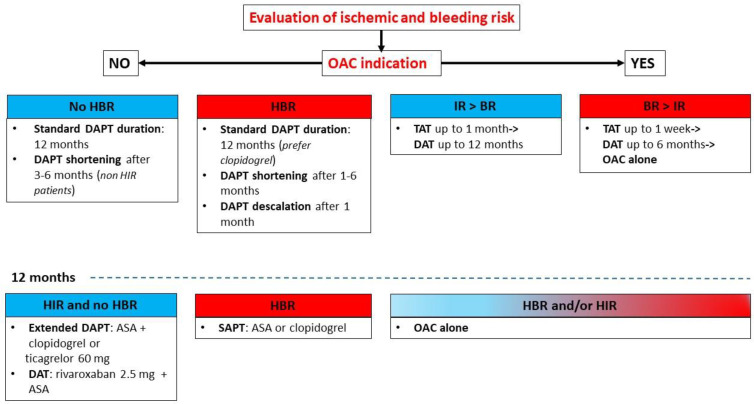
Antithrombotic strategies in elderly patients with ACS undergoing PCI [29,33,34,35,36,37,38,39,45,46,47,48,49,50,51,55,56,57,58,59,64,66,67,69,73,74,75,76,77]. HIR: high ischemic risk, IR: ischemic risk, BR: bleeding risk.

For these reasons, the management of antiplatelet therapy in patients with ACS undergoing PCI remains debated. The evidence so far accumulated for the management of antiplatelets in elderly patients suggests considering clopidogrel in the standard DAPT strategy, evaluating alternative strategies like DAPT shortening and de-escalation, and considering in patients with AF a short course of TAT (1–4 weeks) followed by DAT in order to reduce the risk of bleeding events (Figure 2). Furthermore, additional strategies to reduce the bleeding risk include the use of radial artery access for coronary revascularization, the choice of DES approved for HBR patients with an abbreviated DAPT regimen, the avoidance of routine pretreatment with P2Y12 inhibitors, the careful dosage of anticoagulant doses adjusted to body weight and renal function, the use of proton pump inhibitors, and optimal blood pressure control [91].

New data on antiplatelet drugs in elderly patients are expected from the results of the ongoing PLINY THE ELDER trial (PLatelet INhibition with two different doses of potent P2Y12 inhibitors in THE ELDERly population), which will determine whether a lower dose of ticagrelor (60 mg twice daily) confers non-inferior platelet inhibition compared with the standard dose in the early phase of ACS among elderly patients undergoing PCI [92].

## 8. Conclusions

Elderly patients presenting with ACS and undergoing PCI represent a growing part of our population. The management of antiplatelet therapy in this population is a challenging topic due to the increased risk of both ischemic and bleeding events. During the assessment of elderly patients, clinicians should not worry about chronological age; rather, they should estimate biological age considering concomitant comorbidities. Therefore, a multidisciplinary and tailored approach, considering clinical, anatomical, procedural, and laboratory data, should be used to define the optimal antiplatelet strategy for each patient. Further randomized studies including only elderly patients are needed to define the optimal antithrombotic strategy. 

## Figures and Tables

**Table 1:** Age-specific data in main randomized studies about standard DAPT.

Study	ACS Population (%)	PCI Rate (%)	Elderly Patients (%)	Groups	Follow-Up (m)	Main Results
CURE [29]	NSTE-ACS (100%)	21.2%	>65 y (49.4%)	Clopidogrel vs. placebo	12	Clopidogrel reduced the rate of ischemic events, but with an increase in major bleedings compared with placebo.
TRITON-TIMI 38 [33]	NSTE-ACS (74%), STEMI (26%)	99%	≥75 y (13%)	Prasugrel (10 mg) vs. clopidogrel	15	Prasugrel significantly reduced the rate of ischemic events, but with an increased risk of major bleeding. Elderly patients had no net clinical benefit from prasugrel.
ELDERLY ACS2 [34]	NSTE-ACS (58.8%), STEMI (41.2%)	99.3%	≥75 y (100%)	Prasugrel (5 mg) vs. clopidogrel	12	No significant difference in the composite of ischemic and bleeding events between the two groups.
ISAR-REACT 5 [35,36]	NSTE-ACS (58.9%), STEMI (41.1%)	84%	≥75 y or low body weight (27.5%)	Prasugrel (5 mg) vs. ticagrelor	12	Prasugrel reduced the rate of ischemic events with no significant difference in major bleedings compared with ticagrelor. In elderly or low-body-weight patients, prasugrel reduced the risk of major bleedings with similar efficacy in terms of ischemic events compared with ticagrelor.
PLATO [37]	NSTE-ACS (60.6%), STEMI (38.5%)	65.6%	≥75 y (15.5%)	Ticagrelor vs. clopidogrel	12	Ticagrelor, as compared with clopidogrel, reduced the rate of ischemic events without an increase in the rate of overall major bleeding, but with an increase in the rate of non-CABG-related bleeding. These findings were not found to depend on age.
POPular AGE [38]	NSTE-ACS (100%)	47.3%	≥70 y (100%); ≥75 y (65%)	Ticagrelor vs. clopidogrel	12	Clopidogrel reduced the rate of major and minor bleeding events without an increase in the combined endpoint of ischemic and bleeding events compared with ticagrelor.

m = months, y = years, CABG = coronary artery bypass graft.

**Table 2:** Age-specific data in main randomized studies about abbreviated DAPT strategy.

Study	ACS Presentation (%)	PCI Rate (%)	Elderly Patients (%)	Groups	Follow-Up (m)	Main Results
GLOBAL LEADERS [45,46]	STEMI (13.1%), NSTE-ACS (33.8%)	100%	75 y (16%)	1 m ticagrelor + ASA -> 23 m ticagrelor vs. 12 m ticagrelor/ clopidogrel + ASA -> 12 m ASA	24	Short DAPT was not superior to standard strategy in the prevention of ischemic events, with no difference in major bleedings. These findings were not found to depend on age category.
TWILIGHT [47,48]	NSTE-ACS (64.8%)	100%	≥65 y (52%)	3 m ticagrelor + ASA-> ticagrelor + placebo vs. 12 m ASA + ticagrelor	12	Short DAPT was associated with a lower incidence of clinically relevant bleeding, with no higher risk of ischemic events compared with standard DAPT. These findings were not found to depend on age category.
MASTER DAPT [49]	STEMI (11.7%), NSTE-ACS (36.5%)	100%	≥75 y (69%)	1 m DAPT -> SAPT vs. 3–12 m DAPT -> SAPT	12	Short DAPT was non-inferior for preventing ischemic events and was superior for preventing major or clinically relevant non-major bleeding compared with standard DAPT. These findings were not found to depend on age category
STOPDAPT-2 ACS [50]	STEMI (56.2%), NSTEMI (43.8%)	100%	≥75 y (28.6%)	1 m DAPT with clopidogrel/prasugrel -> 11 m clopidogrel vs. 1 m DAPT with clopidogrel/prasugrel-> 11 m DAPT with clopidogrel	12	Short DAPT did not achieve non-inferiority to 12 months of DAPT in terms of net clinical benefit, with a numerical increase in cardiovascular events. No treatment interaction by age was observed.
ULTIMATE DAPT [51]	STEMI (27.9%), NSTE-ACS (72.1%)	100%	≥65 y (42.1%)	1 m ticagrelor + ASA -> ticagrelor vs. 12 m ticagrelor + ASA	12	Short DAPT resulted in a lower rate of clinically relevant bleeding and a similar rate of ischemic events compared with standard DAPT. Age analysis revealed no influence on bleeding events, but a significant benefit in reducing ischemic events was found in younger patients

m = months, y = years.

**Table 3:** Age-specific data in main randomized studies about de-escalation strategy.

Unguided						
**Study**	**ACS Presentation (%)**	**PCI Rate (%)**	**Elderly Patients (%)**	**Groups**	**Follow-Up** **(m)**	**Main Results**
TALOS-AMI [55]	STEMI (54%), NSTEMI (46%)	100%	≥75 y (27%)	1 m ticagrelor + ASA-> 11 m clopidogrel + ASA vs. 12 m ticagrelor + ASA	12	De-escalation strategy significantly reduced the risk of net clinical events, mainly by reducing the bleeding events. No treatment interaction by age was observed.
Guided						
TROPICAL ACS [56,57]	STEMI (55.7%), NSTEMI (44.3%)	100%	≥70 y (14.2%)	1w ASA + prasugrel -> 1 w ASA + clopidogrel -> 11.5 m ASA + prasugrel/ clopidogrel based on PFT vs. 12 m ASA + prasugrel	12	Guided de-escalation was non-inferior to standard treatment in terms of net clinical benefit. Age analysis revealed a significant clinical benefit in younger patients.
ANTARCTIC [58]	STEMI (34.4%), NSTEMI (65.6%)	100%	≥75 y (100%)	2w ASA + prasugrel 5 mg -> change in P2Y12i based on PFT (prasugrel 5 mg or 10 mg/clopidogrel) vs. ASA + prasugrel	12	No significant difference in the composite endpoint of ischemic events and bleedings between the two groups.
POPULAR GENETIC [59]	STEMI (100%)	100%	≥75 y (14.6%)	ASA + P2Y12i based on genetic test (clopidogrel/prasugrel or ticagrelor) vs. ASA + prasugrel or ticagrelor	12	Guided de-escalation was non-inferior to standard treatment for thrombotic events and resulted in a lower incidence of bleeding. These findings were not found to depend on age category.

m = months, y = years, PFT = platelet function test, P2Y12i = P2Y12 inhibitor.

## Data Availability

Not applicable.
